# Geographical distribution, evaluation of risk of dengue and its relationship with the El Niño Southern Oscillation in an endemic region of Peru between 2004 and 2015

**DOI:** 10.1186/s13104-019-4537-0

**Published:** 2019-08-13

**Authors:** Wilmer Silva-Caso, Walter Espinoza-Espíritu, Jaquelin Espejo-Evaristo, Hugo Carrillo-Ng, Miguel Angel Aguilar-Luis, Luciana Stimmler, Juana del Valle-Mendoza

**Affiliations:** 1 School of Medicine, Research and Innovation Centre of the Faculty of Health Sciences, Universidad Peruana de Ciencias Aplicadas, Av. San Marcos cuadra 2, Chorrillos, Lima, Peru; 2 Centro de Salud Las Palmas – Red de Salud Leoncio Prado – Ministerio de Salud, Huanuco, Peru; 3 Puesto de Salud Tambillo Grande, Red de Salud Leoncio Prado, Ministerio de Salud, Tingo María, Peru; 4 Instituto Superior Tecnológico Público Naranjillo, Tingo María, Peru; 5 Salud Pública y Gestión Sanitaria, Universidad Nacional Hermilio Valdizán, Huanuco, Peru; 6 Puesto de Salud Alto San Juan de Tulumayo - Red de Salud Leoncio Prado – Ministerio de Salud, Huanuco, Peru; 7 Instituto de Investigación Nutricional, Lima, Peru

**Keywords:** Dengue, Epidemiological factors, El Niño Phenomenon

## Abstract

**Objective:**

To determine the geographical distribution and risk stratification of dengue infection in an endemic region of Peru, and its relationship with the presence of El Niño Southern Oscillation (ENSO).

**Results:**

For the analysis, the definition and information about the ENSO events in Peru was obtained from the SENAMHI and IGP reports. The geographical distribution of dengue cases in the territory comprising the 11 districts is homogeneous. There were 1 498 confirmed cases of dengue reported, the highest incidence was determined in Puerto Inca where it reached an incidence of 3210.14/100,000 hab. Of the 11 districts, 2 were classified as a high risk of transmission, 3 as moderate risk, 3 as low risk and in 3 of them the risk of virus transmission could not be determined.

**Electronic supplementary material:**

The online version of this article (10.1186/s13104-019-4537-0) contains supplementary material, which is available to authorized users.

## Introduction

The dengue virus (DENV) is the most frequently reported pathogen responsible for acute febrile illness in Latin America [1]. It is an arthropod-borne virus, that belongs to the Flaviviridae family, which possess four serotypes closely related to each other (DENV-1, DENV-2, DENV3 and DENV-4). It is characterized because it produces a systemic, dynamic, endemic and epidemic disease [2, 3]. In this context, the understanding of the underlying causes that explain the increase in incidence, the risk of transmission and spread of the dengue virus have become a research priority [4–6]. Several factors can influence the dynamic of the transmission of the virus such as environmental factors (climatic), pathogen related factors and the host immune system [3].

In recent years, more importance has been focused on environmental conditions, such as rainfall and temperature, as important determinants of disease transmission [7, 8]. The climatic factor has been extensively linked to changes in the arthropod vector population dynamics. It has been established that the abundance and distribution of the vector is influenced by temperature, humidity and precipitation patterns. In addition, these factors also determine the modulation of the metabolic activity, the egg production and the feeding behavior of the vector [7–9].

One of the main climatic variables manifested in Peru, with potential epidemiological risks for the dengue virus transmission, is the El Niño Southern Oscillation (ENSO). ENSO is the most important climatic cycle that contributes to year-to-year variability in weather, temperatures and the probability of natural events such as heavy rainfall, droughts and storms [10]. These extreme weather conditions may exacerbate or trigger many health risks such an increase in vector-borne diseases, food-borne diseases, malnutrition and disruption of health services [10, 11].

During the 21st century, according to the Oceanic Niño Index (ONI) of the National Oceanic and Atmospheric Administration of the United States of America—NOAA, there have been five El Niño episodes in the Pacific central; three of weak intensity (years 2004–2005, 2006–2007, 2014–2015) and two of moderate intensity (years 2002–2003 and 2009–2010) [12–14].

There are several studies in tropical countries, that establish a relationship in the incidence of arthropod-borne infectious diseases to natural events such as ENSO. In Peru there are only a few reports in coastal areas [15], despite being a country where dengue is an endemic disease.

The objective of this study is to describe the geographical distribution and risk stratification of dengue in an endemic area of central eastern Peru in the department of Huánuco and its relationship with El Niño Southern Oscillation (ENSO).

## Main text

### Materials and method

#### Study population

Observational, descriptive and retrospective study of confirmed cases of dengue between January 1, 2004 and December 31, 2015, registered by the health facilities that correspond to the Executor Unit 403—Leoncio Prado Health Network, reported to the health system surveillance and compiled in a database by the epidemiology unit with public domain results. The cases include the entire population without distinction of age or sex, with dengue confirmed by laboratory according to the operational definitions of the World Health Organization [3].

#### Standardized definitions of ENSO, ONI and Intensity of El Niño

ENSO and ONI are defined according to the National Oceanic and Atmospheric Administration (NOAA). ONI equal to or greater than + 0.5 °C defines El Niño. From the threshold (0.5 °C) it is subdivided into events: weak (with an anomaly of 0.5 to 0.9 °C SST), moderate (1.0 to 1.4 °C), strong (1.5 to 1.9 °C) and very strong (≥ 2.0 °C) [16].

Accumulated rainfall and temperature data:

The accumulated precipitation, maximum temperatures and minimum temperatures data were obtained from the Servicio Nacional de Meteorología e Hidrología del Perú (Senamhi). With the data obtained, annual averages and standard deviation were calculated.

#### Data analysis

The data was sorted by ascending years, the descriptive statistics and the quartile and interquartile values of the incidence of dengue were obtained using the SPSS version 21. The DIVA-GIS software version 7.5.0 was used, which allows locating the geographic coordinates. For georeferencing, the districts were taken as reference and geographical location coordinates were subsequently generated with the Google Maps tool (latitude and longitude).

#### Risk stratification by district—determination of risks levels

The average population per district was taken as a biotic variable. To determine the risk levels, the quotient between number of cases and district inhabitants was considered as recommended by Brito-Hoyos et al. [17]. Districts with an absolute incidence of cases below the 25th percentile were classified as having an unidentifiable risk; those who presented an incidence between the 25th and 50th percentiles were classified as low risk; those that were between the 51st and 75th percentile were classified as moderate risk; and those with an incidence greater than the 75th percentile were classified as high risk districts. Once the classification of the districts was obtained, a risk map was constructed using the DIVA-GIS software version 7.5.0.

### Results

The geographical distribution of dengue cases in the endemic districts of Huamalies, Leoncio Prado and Puerto Inca was homogeneous. There were cases clustered located to the east, in the districts that share boundaries with the department of Ucayali. While the districts located to the west that have a higher altitude above sea level and share boundaries with the Andean regions reported a lower incidence (Additional file 1: Figure S1).

In the 11 districts studied, 1498 confirmed cases of dengue were notified between January 1, 2004 and December 31, 2015. The Rupa-rupa district reported the highest number of cases with a total of 783 (52.27%) cases, followed by Puerto Inca with 281 cases (18.76%) and Jose Crespo y Castillo with 239 cases (15.95%). In relation to the incidence calculated, Puerto Inca reported an incidence of 3210.14 cases per 100,000 population, followed by Rupa-rupa with an incidence of 1286.82 cases per 100,000 population. These two districts were considered as high risk of transmission of the virus (Table 1).

**Table 1:** Percentage and incidence per 100,000 population of confirmed dengue cases and risk stratification by district between 2004 and 2015

Province	District	Cases			Risk level	Inhabitants	Situation
		N	%	Incidence			
Huamalíes	Monzon	24	1.60	107.51	Low	22,323	North
Leoncio Prado	Daniel Alomias Robles	32	2.14	443.71	Low	7212	North
	Jose Crespo y Castillo	239	15.95	675.61	Moderate	35,376	North
	Hermilio Valdizan	2	0.13	49.57	Non-identifiable	4035	North
	Luyando	42	2.80	450.75	Low	9318	North
	Mariano Damaso Beraun	53	3.54	540.95	Moderate	9798	North
	Rupa-Rupa	783	52.27	1286.82	High	60,848	North
Puerto Inca	Codo de Pozuzo	1	0.07	15.44	Non-identifiable	6476	East
	Honoria	40	2.67	660.17	Moderate	6059	East
	Puerto Inca	281	18.76	3210.14	High	8754	East
	Yuyapichis	1	0.07	16.57	Non-identificable	6034	East

The highest number of dengue cases was reported in the 20 to 24 age group, with 211 cases, followed by 181 cases in the 15 to 19 age group. The groups with less cases were 0 to 4 years and older than 60 years (Table 2).

**Table 2:** Distribution of dengue cases according to age group

Age	2004	2005	2006	2007	2008	2009	2010	2011	2012	2013	2014	2015	Total
0–4	1	0	4	0	2	6	4	2	2	0	3	9	33
5–9	7	1	6	0	14	16	7	5	13	3	10	12	94
10–14	9	2	12	3	13	28	30	9	34	6	15	15	176
15–19	9	1	17	3	9	21	16	19	53	9	13	11	181
20–24	11	1	18	1	10	47	32	18	42	6	16	9	211
25–29	5	1	10	2	11	27	26	13	28	8	15	8	154
30–34	8	0	16	2	9	30	26	17	28	6	14	11	167
35–39	2	1	9	4	12	24	21	17	17	3	13	13	136
40–44	2	0	5	2	13	16	10	11	24	9	8	6	106
45–49	2	0	5	0	5	15	9	10	24	3	1	10	84
50–54	2	0	7	0	3	8	12	3	16	5	3	1	60
55–59	0	0	1	0	1	6	6	3	7	1	6	4	35
60–64	0	0	1	0	2	3	4	1	4	0	3	1	19
65–69	0	0	0	0	0	4	1	1	4	0	1	4	15
70–74	0	0	1	0	1	5	2	0	1	0	1	3	14
75–80	0	0	2	0	1	1	3	0	0	0	0	3	10
> 80	0	0	0	0	1	0	0	0	1	0	0	1	3
Total	58	7	114	17	107	257	209	129	298	59	122	121	1498

**Fig. 1:**
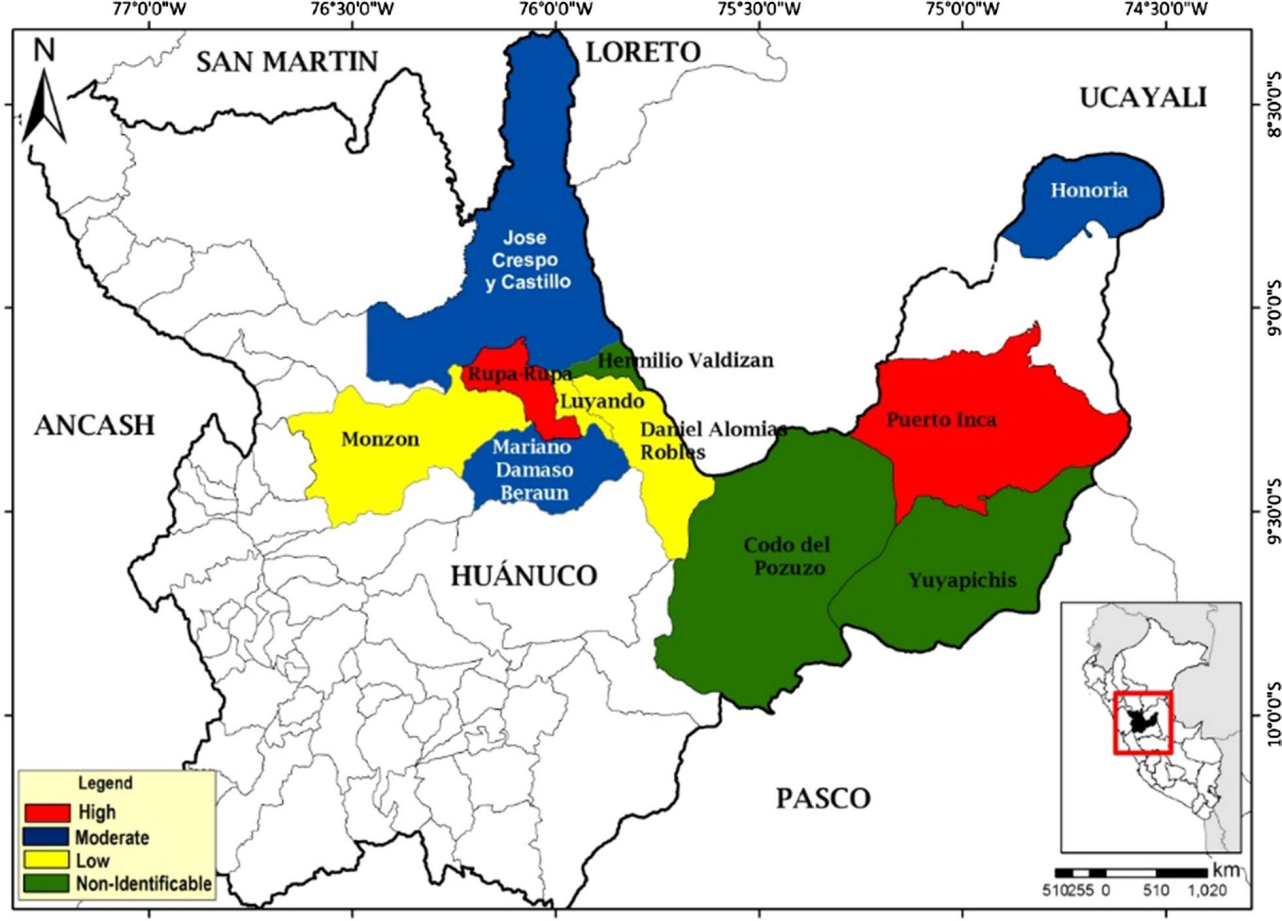
Risk stratification for the transmission of dengue infection

The risk stratification for the transmission of dengue in the 11 districts evaluated is represented by a map. The high risk areas appear in red, moderate risk areas in blue, low risk areas in yellow and undetermined risk areas in green. Two districts were classified as high risk, three as moderate risk, three as low risk and three as undetermined risk (Fig. 1).

The relationship between the dengue annual incidence and the presence of ENSO was established in the years 2004–2005, 2006–2007 and 2014–15, when such phenomenon was classified as low intensity; and in the years 2009–2010 when it was classified as moderate intensity. Our observation indicates that the presence of ENSO of low and moderate intensity was not associated with an increase in the incidence of dengue. On the contrary, in some years, such as 2012 when there was no ENSO reported, the incidence of dengue was higher compared to years in which this phenomenon was reported (Additional file 1: Figure S2).

The values of the annual averages of accumulated precipitation, maximum and minimum temperature of the region remained constant throughout our study. They are not related to the presence or absence of El Niño and are not directly related to the years of high or low incidence of dengue (Additional file 1: Table S1, Figure S3).

### Discussion

The Panamerican Health Organization, estimates that on the incidence of dengue in the Americas was 282.4 cases/100,000 inhabitants during the period between 2010 and 2015 [18]. In Peru, the dengue virus is highly endemic; between 4000 and 29,000 cases per year have been reported during the last 10 years [19, 20]. The trend is towards the increase of transmission since the reinfestation of vectors in the country in the 1980s [21]. We found that in 7 of the 11 districts studied the incidence surpass the average values registered by the PAHO. In these districts, the incidence of dengue is between 450.75/100,000 inhabitants and 3210.14/100,000 inhabitants in the 2004–2015 period.

These findings suggest that there are zones with a greater risk for dengue transmission, which cannot be explained only by meteorological variables. Our study identifies a peak of incidence in 2012, consistent with a study in Peru by Charette et al. which describe a large outbreak of dengue in 2012 that caused more than 10,000 cases and 13 deaths in Ucayali, a region with borders to the East of the territory included in our study. It was suggested that the outbreak was triggered by the introduction of a new virus serotype (DENV-2 in Asia/America) [22]. Our observation indicates that this important outbreak was developed in the absence of the El Niño. The introduction of a new serotype, increased frequency of travel, rapid urbanization and inadequate water management were determinant factors in the spread and transmission of the virus in that year [1, 22]. This suggests that in years when ENSO is absent there may be a high incidence of dengue virus infection, so the presence of this weather phenomenon alone would not be necessary or sufficient to produce an increase in the incidence of dengue in a determined endemic territory.

It is a constant challenge to understand how climate variability and long-term climate change affect the transmission of dengue and other vector-borne diseases [23]. In 2016, Lowe et al. could predict a dengue outbreak using a model that incorporated precipitation, minimum temperature, and Niño3·4 index forecasts. This model accurately predicted the epidemic that occurred in 2010 and the low incidence of dengue in 2013 in the area studied, however it was not decisive for the other years [24]. The main advantage of this tool is that it could provide an anticipated warning of future dengue outbreaks, as well as the timing and magnitude of these events, so preventive measures can be considered.

It is known that vector-borne disease are affected by climatic factors through ecological and biological processes that may favor their expansion [25] but apparently this is not enough to achieve a dramatic increase in the incidence of the disease as other factors would be, for example, the introduction of a new serotype in the case of dengue [22]. In addition to the climatic factor, other studies are investigating important co-factors for dengue transmission such as the entry rate of travelers and the mortality rate of dengue mosquitoes [26], in contrast some studies indicate that the average daily temperature, the variation of temperature and other meteorological variables such as rainfall are more important factors in the current distribution and incidence of dengue [8, 27]. On this point we found no relationship between accumulated rainfall and temperatures with the presence of El Niño and the incidence of dengue. These contradictory results may respond to the fact that daily rainfall totals can be spatially heterogeneous across large geographic territories [28].

Our study shows that meteorological variables such as the ENSO of low intensity and moderate intensity do not correlate with an increase in the incidence of dengue. Regarding the risk of disease due to dengue virus, Colón-Gonzales et al. suggest that future studies should consider other factors along with meteorological variables, such as urbanization and international travel [29].

We conclude that the distribution of dengue virus is homogenous in the endemic region studied. There are zones with a higher incidence that are correlated with a greater risk of dengue transmission and that there is not an increase in the incidence of dengue in the presence of ENSO. Further studies are required to understand the dynamics the dengue transmission and to better characterize the role of the climatic factor.

## Limitations

Our study is limited to a specific geographic region that also does not necessarily represent a significant spatial unit for the dynamics of dengue disease. However, this study gives us the possibility to continue analyzing the geographical and climatic factors involved in the dynamics of dengue transmission at present.

## Additional file

**Additional file 1: Figure S1.** Geographical distribution of the Dengue cases. **Figure S2.** Incidence of Dengue and its relationship with ENSO. Red arrows indicate the occurrence of ENSO. **Figure S3.** Climate Variable/Year. **Table S1.** Climate Variable/Year.

## Data Availability

Abstraction format used in the study and dataset are available and accessible from the corresponding author upon request in the link: https://figshare.com/s/cad05ae27564a8c69351

## Abbreviations

ENSO: El Niño Southern Oscilation
DENV: dengue virus
ONI: Oceanic Niño Index
