# Supplementary material for: Geographical distribution, evaluation of risk of dengue and its relationship with the El Niño Southern Oscillation in an endemic region of Peru between 2004 and 2015
Source: BMC Res Notes. 2019 Aug 13;12:498. doi: 10.1186/s13104-019-4537-0 (PMC6692953; doi:10.1186/s13104-019-4537-0)
Supplement: Supplementary file 1 — Additional file 1: Figure S1. Geographical distribution of the Dengue cases. Figure S2. Incidence of Dengue and its relationship with ENSO. Red arrows indicate the occurrence of ENSO. Figure S3. Climate Variable/Year. Table S1. Climate Variable/Year. [file 13104_2019_4537_MOESM1_ESM.docx]

**
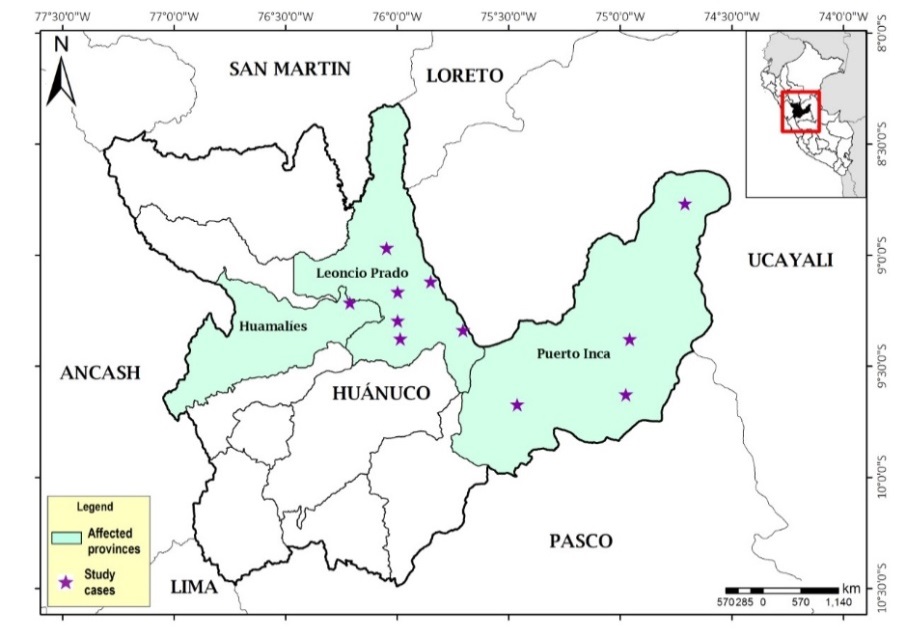
Figure S1. Geographical distribution of the Dengue cases**


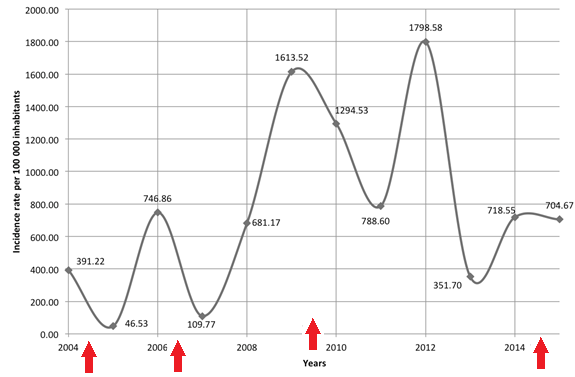
**Figure S2. Incidence of Dengue and its relationship with ENSO. Red arrows indicate the occurrence of ENSO**

**Figure S3: Climate Variable / Year**

*El Niño event of mild intensity

**El Niño event of moderate intensity

**Table S1: Climate Variable / Year**

| **Climate Variable / Year** | 2004* | 2005* | 2006* | 2007* | 2008 | 2009** | 2010** | 2011 | 2012 | 2013 | 2014* | 2015* |
| --- | --- | --- | --- | --- | --- | --- | --- | --- | --- | --- | --- | --- |
| Accumulated Precipitation (mm) | 8.24 SD 13.91 | 8.07 SD 16.09 | 9.69 SD 16.90 | 8.66 SD 13.78 | 8.08 SD 13.33 | 9.89 SD 13.71 | 8.32 SD 13.98 | 9.84 SD 15.57 | 8.73 SD 14.50 | 10.26 SD 18.55 | 11.6 SD 19.72 | 8.62 SD 16.40 |
| Maximum Temperature (° C) | 29.4 SD 2.07 | 30.09 SD 1.93 | 29.79 SD 1.95 | 29.9 SD 1.92 | 29.49 SD 2.10 | 29.63 SD 1.99 | 30.45 SD 2.16 | 29.67 SD 2.40 | 30.26 SD 2.16 | 30.12 SD 2.18 | 29.86 SD 2.5 | 30.37 SD 2.05 |
| Minimum Temperature (° C) | 20.26 SD 1.15 | 20.3 SD 1.17 | 20.07 SD 1.06 | 20.28 SD 1.04 | 20.17 SD 0.95 | 20.49 SD 0.80 | 20.43 SD 1.08 | 20.2 SD 0.97 | 20.23 SD 1.08 | 20.55 SD 0.96 | 20.94 SD 0.9 | 20.75 SD 0.80 |

*El Niño event of mild intensity

**El Niño event of moderate intensity

Sd = standard deviation
